# Observations on the Utility of Filing the Teeth

**Published:** 1844-09

**Authors:** John Harris

**Affiliations:** Georgetown, Ky.


### ARTICLE III.

### *Observations on the Utility of Filing the Teeth.* By JOHN HARRIS, M. D., D. D. S., of Georgetown, Ky.

FILING the teeth is one of the most important and valuable resources of the dental art; it is one that has stood the test of experience, and is of such acknowledged utility, as to constitute of itself, in the treatment of superficial caries on the lateral surfaces of the teeth, one of the most valuable operations that can be performed on these organs. And, even after caries of the teeth, in the localities just mentioned, has progressed so far as to render its removal, by this means, impracticable or improper, the use of the file, in most cases, is still necessary, in order to the successful employment of other remedial agents. But in either case, a failure to accomplish the object for which it is used, would only be equivalent to doing nothing at all.

The use of the file then, may very justly be considered a *sine qua non*, for the removal of superficial caries from the sides of the teeth which come in contact with each other, as can be attested by thousands of living witnesses, and in preparing the way, in deep-seated caries, for the thorough removal of the disease, and the filling, successfully, of the cavity thus formed.

In a paper written by myself, some eleven or twelve years ago, upon this subject, I contended, that filing the teeth was not necessarily productive of caries, and my subsequent experience and observation have only tended to confirm the correctness of the opinion which I then advanced, and I cherish the belief that this opinion, will not, at this time, conflict with the views of the more enlightened of my professional brethren.

But when reference is had to the physical peculiarities of the teeth, it will at once be perceived, that they present a strange departure from the laws that govern and control all other parts of the body—that these organs, when diseased, can only be restored to health and usefulness by art, unaided by the sanitary powers of nature. Hence it is, that most of the operations upon them, will not, like those in general surgery, admit of mediocrity in their performance.

Empiricism is common in all professions, but in no one is it so much to be deprecated and feared as in the medical, and in no one branch of which, more than in that of dental surgery. However unskilfully and awkwardly an operation in general surgery may be performed, if the patient survives, the ignorance or lack of skill on the part of the surgeon, instead of being exposed to merited censure, is put down to his credit; but any deficiency in the qualifications of the dentist, soon becomes manifest to the patient, who visits upon the author of his disappointed hopes and aggravated condition, a justly deserved indignation. But notwithstanding his shaken confidence in the utility or remedial abilities of the art, he may be induced again and again to seek its aid by calling on other dentists, and, perhaps, without any better success, and, finally, losing all confidence in the resources of the profession, he considers his case hopeless, and gives himself up to despair. His prejudices thus contracted, are made known to, and exert an influence upon all his friends and acquaintances, many of whom, perhaps, for want of confidence thus engendered, and fear of sharing a similar fate, prefer the loss of their teeth, rather than submit to operations, without any prospect of obtaining relief.

When reference is had to the character and qualifications of a large majority of the persons engaged in the practice of dental surgery in the western states, the cause of the prejudices here referred to, are at once explained. But from what has already been done, and from the efforts that are now being made in our Atlantic cities and other places to elevate and give character and consequence to the science and practice of this branch of the healing art, we have reason to believe, that the time is not distant, when only such as have well grounded claims to skill, will be permitted to exercise its duties.

But I pass on. The fact that the crowns of the teeth are covered with enamel, is alone sufficient evidence of its importance and utility in shielding and protecting the bony structure, which it envelopes, from mechanical and morbid influences, so that it would seem that its removal or loss would necessarily expose the organs to certain destruction. But we have satisfactory evidence, that teeth, after having suffered the loss of large portions of the enamel, have been restored to health and preserved for many years, and often through life.

The rapidity with which caries of the teeth progresses, after the exposure of the bone, by the loss of the enamel, depends upon the physical peculiarities of the organs, and upon local and constitutional influences; hence the difficulty, and oftentimes impossibility of obtaining the object for which dental operations are instituted, while such influences are suffered to exist. If special regard is not had to the curative indications, most, if not all of the operations upon the teeth, which have for their object their ultimate preservation, are sure, to a greater or less extent, to augment all of the previously existing local affections, by increasing the irritability of the parts, and by rendering them more susceptible of being acted upon both by local and constitutional causes.

Without indulging in further prefatory remarks, I shall proceed to notice more particularly the subject under consideration. And I would observe, firstly, that an experience obtained from twenty-three years' constant practice, has fully convinced me, not only of the propriety, but of the absolute necessity, in the treatment of caries of the lateral surfaces of the teeth, of employing the file. There is no instrument so well adapted as this for the removal of the disease when situated in these parts of the teeth, and especially when the organs are in close promixity with each other, or for the removal of rough and weakened edges of the enamel in deep-seated caries, and for making sufficient space or room for the removal of the diseased or unsound parts preparatory to plugging.

It may be laid down as a rule, from which exceptions should never be taken, that the file should not be used, while the teeth or their contiguous parts are suffering general or local, acute or chronic, inflammation. Therefore, when this is the case, the treatment of the general and local affections should be precedaneous to the operation of filing. Upon the subjugation of all the acute or chronic diseases of the mouth, the success of the dentist in the treatment of affections of the teeth, calling for the employment of the file, greatly depends. As much importance, therefore, is to be attached to an enlightened and discriminating judgment, as to tact in the performance of the operation.

In fact, the removal of all local causes of irritation, such as all dead roots of teeth, teeth occasioning alveolar abscesses, or such as exert a morbid influence upon the surrounding parts, and all depositions of salivary calculus or other foreign matter, should always precede all other operations upon these organs.

The length of time necessary for the restoration of the parts contiguous to the teeth to a healthy condition, may vary from a few days, or weeks, to months, depending upon the nature and extent of the disease in them, the general health of the patient, and the constitutional as well as local treatment to which they are subjected. The frequent failures in accomplishing the object for which dental operations are instituted, are, in most instances, the result of the ignorance of the practitioner, or the want of a correct knowledge of the nature, cause and curative indications of the disease he attempts to treat.

But, in assuming the position, that the filing of the teeth, does not, of necessity, cause them to decay, it is by no means to be inferred, that the operation can in all cases, and under all circumstances, be performed with advantage or even impunity. By no means, its effects, like those of most other operations upon the teeth, when the curative indications are disregarded, or not properly carried out, are never passive. The employment of the file at an improper time, and in an improper manner, increases the liability of the teeth to decay, and augments the irritability of all the parts adjacent to them, and consequently their susceptibility of being acted upon by local and constitutional causes.

This view of the subject, taken in connection with the fact, that comparatively few of those engaged in the practice of this branch of surgery, are properly qualified for it, satisfactorily accounts for the pernicious effects that so frequently result from the use of the instrument in question, and for the wide-spread and deep-rooted prejudice that has obtained against its employment.

The principal, and I believe only, objection urged against filing the teeth, is based upon the erroneous belief, that the loss of any part of the enamel of these organs, must necessarily result in their destruction. But if this be true, why is it, as I have on another occasion asked, that the negroes of Abyssinia have such sound teeth as they are represented to have, since it has long been a custom with them, to file all their front teeth to points, so as to make them resemble the teeth of a saw or those of carnivorous animals. Of course, large portions of the enamel and considerable of the bony structure, must be removed in the operation, and yet we are credibly informed that their teeth seldom decay. The same may be said of the Brahmins of India, who, from remote ages, have been in the habit of using the file, principally, I believe, for separating their teeth, and they too, are noted for having fine teeth. I might refer to the people of other countries, with whom the same practice has long had an existence, but it is not necessary to go abroad for proof, when we have such an abundance of it at home, to establish the propriety and absolute necessity of the practice I am now advocating.

With the people just referred to, it is evident that they file principally for the purpose of ornamenting their teeth, but with us, only as a remedial agent in the treatment of their diseases. The reason why their teeth are not so subject to disease as are those of the inhabitants of luxurious and civilized countries, is attributable to the difference in their habits of life, modes of living, and the absence of the causes productive of the various diseases peculiar to civilization and refinement.

But notwithstanding the utility and value of the operation, the filing of the teeth may be regarded as a predisposing cause of caries. But if this be true, it may be asked, why file at all? I answer, in this country, owing to the prevalence of the immediate or direct cause of caries, the operation is only performed as remedial, for the purpose of removing *actual* disease, or as preparatory to plugging. It does not, of necessity follow, that caries of the teeth, after having been judiciously removed or treated, although the organs be predisposed to the disease, should ever again occur. The general system often escapes the development of disease to which it is predisposed through life, so also do the teeth. If the operation be properly performed, and the filed surfaces kept thoroughly clean, a recurrence of the disease, notwithstanding the increased predisposition thus induced, will never again take place. The immediate cause of dental caries being the contact of corrosive agents with the teeth, the necessity for this precaution is obvious. The bony structure of these organs is more easily acted upon by such causes, than the enamel, and for this reason, when it becomes necessary to expose it, for the removal of disease, with a file, it should be done in such a way as to admit of its being kept thoroughly and constantly clean, so that if it afterwards becomes carious, it will be owing altogether to the inattention of the patient. In view of this, whenever it becomes necessary to file the teeth, whether for the complete removal of caries, or as only preparatory to plugging, we should always impress upon our patient the importance of attending to this matter, of cleaning the surfaces thus operated upon, at least three or four times every day. The future preservation of the organs, and especially such as are of a soft or chalky texture, for they are then by far more easily acted upon by decomposing agents than when hard, will depend upon the constant and regular observance of this precaution.

The cases requiring the use of the file vary so much that it would be difficult to lay down precise directions with regard to the extent to which the operation should always be carried. This must be determined by the judgment of the operator.

The object for which the operation is performed, may be defeated either by filing too much or too little. Either extreme should be avoided, but I am of the opinion, that by far the greater number of unsuccessful results are attributable, rather to the too moderate than to the too great use of this instrument, and especially where the circumstances of the case have nothing to do in determining such results.

But it is not my object to describe the manner in which teeth should be filed, but merely to offer a few general remarks on the advantages that result from it when the operation is judiciously performed, and to show, that it is from the abuses of the use of the file, in the hands of ignorant and inexperienced practitioners, that its merits have been so frequently erroneously estimated.

It will be perceived from the foregoing remarks, that its utility depends upon carrying out all the curative indications, that it should never be resorted to except in the absence of disease in the parts with which these organs are immediately connected. Therefore, to estimate the merits of the operation, correctly, we should know all the circumstances under which it has been performed, the competency of the operator, and whether he was permitted the free exercise of his judgment.

The dentist is often called upon to render his services, where, from the timidity or ignorance of his patient, he is, if he consents to operate at all, so restricted in the application of his remedies, that but little, if anything more than temporary relief can be afforded. And cases may occasionally occur, in which, from unforeseen circumstances, even after the most skilful management, the dentist may be disappointed in his expectations, and fail in the attainment of the object for which his services were solicited.

But numerous as are the diseases of the teeth and those of the parts immediately connected with them, and formidable as they often become, there are comparatively few cases, in which the resources of dental surgery, in its present improved state, are not amply sufficient to afford more or less benefit, even if the desired object be not fully attained.

